# Tracheostomy care for adults and the elderly in the home environment: a scoping review

**DOI:** 10.1590/1980-220X-REEUSP-2024-0028en

**Published:** 2024-08-02

**Authors:** Aldenora Laísa Paiva de Carvalho Cordeiro, Jade Alycia Ribeiro e Santos, Ana Clara Leite Barroso, Miguir Terezinha Vieccelli Donoso, Luciana Regina Ferreira Pereira da Mata, Tânia Couto Machado Chianca

**Affiliations:** 1 Universidade Federal de Minas Gerais, Escola de Enfermagem, Departamento de Enfermagem Básica, Belo Horizonte, MG, Brazil.; 2 Universidade Federal de Minas Gerais, Escola de Enfermagem, Belo Horizonte, MG, Brazil.

**Keywords:** Tracheostomy, Home Nursing, Patient Discharge, Nursing, Traqueostomía, Atención Domiciliaria de Salud, Alta del Paciente, Enfermería

## Abstract

**Objective:**

To map out scientific knowledge regarding tracheostomy care for adults and the elderly carried out by individuals, famies or caregivers in home environments.

**Method:**

Scoping review, conducted in February 2023, according to the methodology of the Joanna Briggs Institute. The guiding question was: what and how should adult/elderly tracheostomy care be carried out by the individual/family/caregiver in the home environment? Studies published in Portuguese, English and Spanish were considered. The databases consulted were: Lilacs; Medline, via PubMed; Cinahl; Cochrane Library; PEDro; Embase; Scopus and Web of Science.

**Results:**

2158 articles were identified, of which 81 were read in full and 14 included in the review. The main types of care identified included psychobiological needs: airway suction, changing the tracheostomy attachment, cleaning the endocannula and sanitizing the peristomal skin. As for psychosocial needs, incentives for communication and autonomy were identified. There were no recommendations for care related to psychospiritual needs.

**Conclusion:**

The findings prioritize biological care, few studies have detailed how to carry out such care at home.

## RESUMEN

**Objetivo:**

Mapear el conocimiento científico sobre cuidados de traqueostomía para adultos y ancianos realizados por individuos, familias o cuidadores en el entorno domiciliario.

**Método:**

Revisión exploratoria, realizada en febrero de 2023, según la metodología del Instituto Joanna Briggs. La pregunta-guía fue: ¿qué y cómo debe ser realizado el cuidado de la traqueostomía del adulto/anciano por el individuo/familia/cuidador en el ambiente domiciliario? Se consideraron estudios publicados en portugués, inglés y español. Las bases de datos consultadas fueron: Lilacs; Medline, vía PubMed; Cinahl; Cochrane Library; PEDro; Embase; Scopus y Web of Science.

**Resultados:**

Se identificaron 2158 artículos, de los cuales 81 fueron leídos en su totalidad y 14 incluidos en la revisión. Los principales tipos de cuidados identificados incluyeron necesidades psicobiológicas: succión de la vía aérea, cambio del accesorio de traqueostomía, limpieza de la endocánula e higienización de la piel periestomal. En cuanto a las necesidades psicosociales, se identificaron incentivos para la comunicación y la autonomía. No hubo recomendaciones para los cuidados relacionados con las necesidades psicoespirituales.

**Conclusión:**

Los hallazgos priorizan los cuidados biológicos y pocos estudios han detallado cómo llevar a cabo dichos cuidados en el domicilio.

## INTRODUCTION

Tracheostomy is a surgical procedure commonly performed in hospitals, consisting in the opening of the anterior wall of the neck and trachea, where a cannula is inserted to allow communication with the external environment and ensure that the airways remain permeable^(1)^. It is commonly indicated for individuals who need ventilatory support and to reverse prolonged or permanent upper airway obstruction^(2)^.

Tracheostomized individuals risk a number of biological and psychosocial challenges, especially on discharge from hospital^(1)^. The lack of guidance on care at home increases the chance of complications that can result in readmissions^(1,2)^.

Early discharge and failure to train the patient and family/caregiver in home care have been recognized as one of the main risk factors for hospital readmissions of individuals with tracheostomies^(3,4)^. Given that in the post-operative period and at hospital discharge, care is often offered in a fragmented way, support and instructions on how to carry out home care are essential^(1)^.

Hospital discharge planning for an effective transition of care ensures the continuity and safety of the tracheostomized patient, preventing critical incidents and rehospitalizations^(2)^. This planning should be understood as a continuous process that begins at hospital admission and should involve the individual-family-community triad^(1,3)^.

However, although scientific knowledge^(1–4)^ reinforces the importance and need for teaching and counseling the person and/or family/caregiver, it is not clear what and how tracheostomy care for adults/elderly people should be carried out in the context of the home environment. The American Association for Respiratory Care (AARC) published in 2022 the Clinical Practice Guidelines^(5)^ on airway aspiration, but its publications still lack guidelines that address the specifics of tracheostomy care at home.

In a preliminary search carried out in January 2023 on the Medical Literature Analysis and Retrieval System Online (Medline), the Cochrane Database of Systematic Reviews and the Joanna Briggs Institute (JBI) Evidence Synthesis, no review studies were found addressing home care. Only one scoping review^(2)^ was found; however, the focus of the nursing interventions mapped was self-care with the tracheostomy, with no description of how to carry out care in the home environment^(2)^, which justifies the proposition of this study. Thus, the aim of this scoping review was to map the scientific knowledge on tracheostomy care for adults and the elderly carried out by the individual, family or caregiver in the home environment. The aim of this review is to clarify what care is needed and how it should be carried out.

## METHOD

### Type of Study

This is a scoping review. The protocol for this review was registered on the Open Science Framework (OSF) platform^(6)^ and followed the methodology proposed by the JBI, described in the JBI Reviewer’s Manual^(7)^. The recommendations of the Preferred Reporting Items for Systematic Reviews and Meta-Analyses for Scoping Reviews (PRISMA-ScR)^(8)^, which is specific to scoping reviews, were followed for writing.

### Guiding Question

The guiding question was structured according to the mnemonic “PCC”, from Population, Concept and Context^(7)^: P- adult and elderly tracheostomized patients; C – care provided by the patient themselves, family or caregiver; C- home environment. Thus, the guiding question of this review was: “what and how should tracheostomy care be carried out in adults/elderly patients by the individual/family/caregiver in the home environment?”

The concept and context “care carried out by the patient themselves or family/caregiver in the home environment” is anchored in Medline’s Medical Subject Headings (MeSH) definition for the term “Home Nursing”, which refers to nursing care provided to a person at home, which can be carried out by the patient themselves, family, friend or a properly trained caregiver.

### Eligibility Criteria

The selection criteria were defined based on the guiding question, considering the PCC strategy. Studies with the following characteristics were considered: a) in terms of population – studies carried out with individuals with a permanent or temporary tracheostomy, aged 18 or over; b) in terms of concept - studies that assessed or described care that could be carried out by the individual themselves or their family/caregiver; c) in terms of context - studies that included care carried out in the home environment.

Studies published in Portuguese, English and Spanish were included; primary research with quantitative, qualitative and mixed methods designs, review studies and clinical opinion studies. No time limit was set for the publication of the included studies. Studies that did not present the scenario of tracheostomy care were excluded.

### Search Strategy and Selection of Studies

The search strategy was designed by a professional librarian and the following databases were considered: Latin American and Caribbean Health Sciences Literature (Lilacs); Medline, via PubMed; Cumulative Index to Nursing and Allied Health Literature (Cinahl), Cochrane Library, Google Schoolar, Physiotherapy Evidence Database (PEDro), Embase, Scopus and Web of Science. The last three databases were accessed through the CAPES (Coordination for the Improvement of Higher Education Personnel) portal in Brazil. Sets of controlled and uncontrolled terms related to population (P), concept (C) and context (C) were organized. A search was also carried out in the gray literature using the following databases: the Capes Catalogue of Theses and Dissertations; the Brazilian Portal of Publications and Scientific Data in Open Access (OASISBR) and OpenGrey.

Chart 1 below shows the search strategy used for each database.

**Chart 1:** Search strategies applied to each database, Belo Horizonte, MG, Brazil, 2023.

DATABASE	STRATEGY
LILACS	(traqueostomia OR tracheostomy OR traqueostomía OR trachéostomie) AND (“Assistência Domiciliar” OR “Home Nursing” OR “Atención Domiciliaria de Salud” OR “Soins à domicile” OR “Ambiente Domiciliar” OR “Home Environment” OR “Ambiente en el Hogar” OR “Enfermagem Domiciliar” OR “Home Health Nursing” OR “Cuidados de Enfermería en el Hogar” OR “Soins infirmiers à domicile” OR “Home Care”) AND ( db:(“LILACS” OR “BDENF” OR “IBECS” OR “BINACIS” OR “campusvirtualsp_brasil” OR “CUMED” OR “INDEXPSI” OR “MedCarib” OR “SES-SP” OR “SMS-SP” OR “SOF” OR “colecionaSUS”))
MEDLINE VIA PUBMED	(Tracheostomy) AND (“Home Nursing” OR “Home Environment” OR “Home Health Nursing” OR “Home Care”)
CINAHL	(Tracheostomy) AND (“Home Nursing” OR “Home Environment” OR “Home Health Nursing” OR “Home Care”)
COCHRANE	(Tracheostomy) AND (“Home Nursing” OR “Home Environment” OR “Home Health Nursing” OR “Home Care”)
GOOGLE SCHOLAR	(Tracheostomy OR Traqueostomia) AND (“Home Nursing” OR “Assistência Domiciliar”)
PEDro	(Tracheostomy)
EMBASE (Via Portal Capes)	(Tracheostomy) AND (‘Home Care’ OR ‘Home Environment’)
SCOPUS (Via Portal Capes)	(Tracheostomy) AND (“Home Nursing” OR “Home Environment” OR “Home Health Nursing” OR “Home Care”)
WEB OF SCIENCE (Via Portal Capes)	(Tracheostomy) AND (“Home Nursing” OR “Home Environment” OR “Home Health Nursing” OR “Home Care”)
**GREY LITERATURE**	**STRATEGY**
CAPES catalog of theses and dissertations	(Traqueostomia) AND (“Assistência Domiciliar”)
OASISBR	(Traqueostomia) AND (“Assistência Domiciliar”)
OpenGrey	(Tracheostomy) AND (“Home Nursing”)

The search in the databases was carried out independently by two researchers in February 2023. Pre-selection consisted of reading the titles and abstracts, taking into account the eligibility criteria.

The Rayyan Qatar Computing Research Institute (Rayyan QCRI) review software was used. This program allowed the researchers to carry out the selection of studies in a systematic and rapid way, as well as excluding duplicates and blinding the researchers, which ensured reliability and methodological accuracy for this stage^(9)^.

After pre-selection, the articles were read in full by two researchers independently. In addition, there was a hand search of the reference list of the articles selected for full reading. A third researcher was needed in cases where there was disagreement between the other two researchers. Disagreements were resolved by discussion between the three researchers until a consensus was reached.

### Data Extraction and Analysis

Data extraction was carried out independently by two researchers in order to reduce the risk of measurement bias. The data extraction tool was designed by the researchers and included the following data: author(s), year of publication, origin of the study, objectives, population and sample size, type of study, results and details of these, main findings related to the scoping review question. When necessary, the authors of the papers were contacted to gather information and details about the data.

In the second stage, the data on care was grouped and summarized according to the categories of basic human needs^(10)^ – psychobiological, psychosocial and psychospiritual. All the researchers took part in interpreting and summarizing the data. The data was presented in tabular form and analyzed in narrative form.

## RESULT

**Figure 1:**
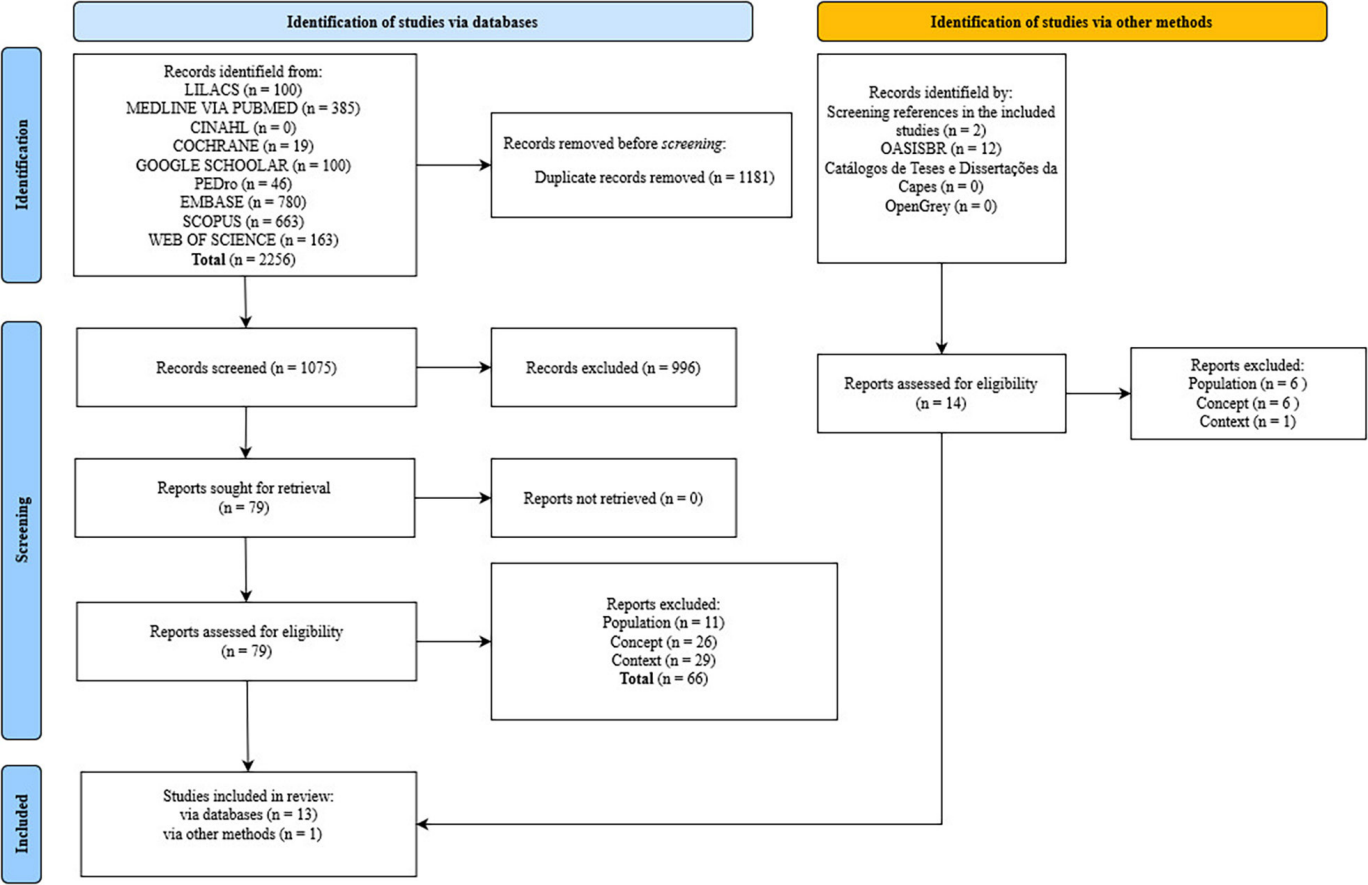
PRISMA-ScR flowchart for selecting publications – Belo Horizonte, MG, Brazil, 2024.

A total of 2,256 articles were identified in the databases and 14 studies were retrieved by manually searching the references of the included studies and the gray literature, as shown in Figure 1. After removing duplicates (n = 1181), 996 studies were excluded during the reading of the abstracts because they did not answer the review question, as they dealt with a population under the age of 18 (n = 475), or did not fit the context and concept established in the research eligibility criteria (n = 521). A total of 93 articles were read, of which 14 made up the sample for this scoping review.

Figure 1 illustrates the flow of the search and selection process for the articles included in the scoping review.

The period of publication of the studies ranged from 1985 to 2022 and, in terms of language, 11 (79%) were published in English, one (7%) in Spanish and two (14%) in Portuguese. As for the location of the studies, three (21.4%) were carried out in the United States; two (21.4%) in Iran; two (21.4%) in Türkiye; two (21.4%) in Brazil; one (7.1%) in England, Chile, New Zealand, Belgium and India. Chart 2 shows the characterization of the studies.

**Chart 2:** Characterization of the studies included in the scoping review. Belo Horizonte, MG, Brazil, 2023. Source: Research data, 2023.

Title	Year/country	Aim	Type of study	Main results
[E1] A systematic review of patient and caregiver experiences with a tracheostomy^(1)^.	2018, New Zealand.	To understand current knowledge related to the experience and quality of life of adults living with a tracheostomy and their caregivers, in order to improve care.	Systematic review.	Five main themes emerged from the research: (1) care, support and management of a tracheostomy; (2) speech and communication; (3) well-being and quality of life; (4) disfigurement and body image; and (5) stigma and social isolation.
[E2] Descripción y manejo del paciente traqueostomizado en Hospitalización Domiciliaria: experiencia en el Complejo Asistencial Doctor Sótero del Río (Description and management of the tracheostomized patient in home hospitalization: experience in the Complejo Asistencial Doctor Sótero del Río.)^(11)^.	2022, Chile.	To describe the profile and management of patients with tracheostomies who are hospitalized at home.	Descriptive, retrospective.	The objective of teaching tracheostomy management and education was applied to 87 of the 96 participating patients and covered general tracheostomy and stoma care, knowledge of the device and management of emergencies and decannulation situations.
[E3] Cuidados para a prevenção de complicações em pacientes traqueostomizados (Care to prevent complications in tracheostomized patients^(12)^.	2019, Brazil.	To analyze the evidence of care for the prevention of complications in tracheostomized patients.	Integrative review.	Factors related to endotracheal suction, tube and skin cleanliness and health education were the main strategies for minimizing the risk of complications.
[E4] As necessidades de aprendizagem dos pacientes laringectomizados (The learning needs of laryngectomized patients)^13)^.	1992, Brazil.	Reporting on the learning needs of laryngectomized patients treated at a university hospital.	Qualitative, participant observation.	The learning needs identified refer to the acquisition of information and skills related to: the function of the larynx, the disease, the objectives and consequences of surgery and treatments; the possibility of carrying out professional, leisure and sexual activities; the possibility of training the esophageal voice; altered body image; and the development of skills for self-care with the tracheostomy.
[E5] Tracheostomy care education and its effect on knowledge and burden of caregivers of elderly patients: a quasi-experimental study^(14)^.	2019, Türkiye.	To investigate the effect of tracheostomy management training for caregivers of elderly patients on caregivers’ levels of burden and knowledge.	Quasi-experimental.	Training increased the caregiver’s level of knowledge and reduced the burden of care. Attitudes and skills to be acquired should be explained in a language understandable to patients and caregivers and taught by demonstration methods during the hospitalization period.
[E6] Developing the tracheostomy care anxiety relief through education and support (T-CARES) program^(15)^.	2014, United States.	To describe the development and results of the T-CARES program, developed in response to the high readmission rates of head and neck cancer patients discharged with a tracheostomy.	Quasi-experimental.	The T-CARES program shows promise in reducing anxiety and increasing aspiration competence in home caregivers of a family member with a new tracheostomy.
[E7] Gaining experience over time: The family caregivers’ perception of patients with a tracheostomy in home care^(16).^	2021, Iran.	To explain the experiences of caregivers and family members of patients with tracheostomies about caring for the patient at home.	Qualitative, content analysis.	The categories of experiences identified in the study include: need for training; need to receive care support; care challenges; burden; experience, hope and inner satisfaction.
[E8] Is disinfection of mechanical ventilation tubing needed at home^(17)^?	2006, Belgium.	To determine the level of cleanliness and sterility of the home ventilation circuit (HVC) in patients on home ventilation and the efficiency of the recommended HVC cleaning protocol.	Experimental.	Recommendations such as using a dishwasher twice a month to wash all parts of the tubes, circuits and ventilation masks, and cleaning tracheostomy tubes with hydrogen peroxide or through a hot bath with a brush and detergent were presented. Decontamination is only indicated when the tube is visually dirty and/or when tracheostomized patients are susceptible to respiratory tract infections.
[E9] Caring for patients with a tracheostomy at home: a descriptive, cross-sectional study to evaluate health care practices and caregiver burden^(18)^.	2019, Türkiye.	To determine the health care practices and burden of family caregivers of tracheostomy patients living at home.	Descriptive, cross-sectional.	The care behavior of caregivers was assessed in the following categories: knowledge of emergency care, number of instruments for care, frequency of home visits, experience in emergency situations in the last six months and the patient’s activities of daily living.
[E10] Home care nursing advice for patients with head and neck cancer in India^(19)^.	2006, India.	Instructing patients, family members and caregivers on the procedures and care required for the mouth, tracheostomy and nasogastric tube.	Clinical opinion.	Tracheostomy care includes changing and cleaning the inner tube; cleaning around the tracheostomy site and changing dressings daily; looking for signs of infection in the mouth and stoma area; as well as preparing solutions to sanitize the structures involved in the device.
[E11] Long-term care of the patient with a tracheostomy^(20)^.	2005, United States.	To discuss the components of a long-term tracheostomy treatment program.	Clinical opinion.	Patient education should begin before tracheostomy insertion and include: basic airway anatomy; justification for tracheostomy use; description of tube operation; signs and symptoms of respiratory distress; suctioning technique; tube cleaning and maintenance; stoma site assessment and cleaning; cardiopulmonary resuscitation; emergency decannulation and reinsertion procedures; tube change procedure; use of equipment and supplies.
[E12] Tracheostomy selfcare: The Nottingham System^(21)^.	1992, England.	To describe the Nottingham System, which aims to teach tracheostomy self-care in a step-by-step manner.	Descriptive.	Care is described as: recognizing the need to clean and change the tube; removing, cleaning and inserting the inner tube; preparing the equipment for changing the outer tube; applying tapes; removing, cleaning and inserting the outer tube; skin care.
[E13] Effect of videotape for home instruction on the quality of life of tracheostomy patients: a randomized clinical trial^(22)^.	2015, Iran.	Verifying the effect of instructional videos on the quality of life of people with tracheostomies.	Quasi-experimental.	The daily care presented was: bathing, shaving, suctioning, dressing the peristomal skin, cleaning the tracheostomy tube, knowing how to identify symptoms of infection at the tracheostomy site, knowing how to communicate with other people and how to present in public.
[E14] Helping a laryngectomy patient go home^(23)^.	1985, United States.	To describe care for laryngectomized patients, ranging from communication to teaching self-care.	Clinical opinion.	Care aimed at helping patients regain independence and autonomy was described, such as communication training, daily cannula removal and cleaning, nutritional counseling and other self-care procedures.

Care was grouped together according to the basic human needs (BHN) proposed by Wanda Horta (1979)^(10)^. Care related to psychobiological needs was mentioned in all the studies in the sample, with the following being highlighted: aspiration of the airways (nine/ 64%); cleaning the stoma and endocannula (12/ 85%); and changing dressings and fastening laces (nine/ 64%). Among the 14 studies, seven (50%) also described care that meets psychosocial needs, with emphasis on re-establishing the patient’s oral communication skills by learning to use the esophageal voice and other speech aids. The need for the patient’s social reintegration, considering their autonomy and support from family and other social groups, was present in two (14%) studies. Care related to psychospiritual needs was not identified.

Chart 3 shows the care identified in the literature grouped into BHN, as well as recommendations on how to carry out this care.

**Chart 3:** Summary of the articles included on tracheostomy care for adults and the elderly in the home environment (n = 14). Belo Horizonte, MG, Brazil, 2023. Source: Research data, 2023.

BHN	Tracheostomy care in the home environment	
	Care	Recommendations on how to carry out care
**Psychobiologic**	Aspiration of secretions^(1,11,12,14,15,18,20,22,23)^.	– Use aseptic technique^(11)^. – Pre-oxygenate the patient^(18)^. – Use vacuum pressure between 100 and 150 mmHg^(11,12)^. – Perform the procedure within 10 to 15 seconds^(11,12,23)^. – Instill saline solution^(23)^. – Use a portable aspirator^(1)^. – Perform the procedure daily, 1 to 5 times a day^(18)^.
	Stoma care: cleaning and changing dressings^(11-16,18-23)^.	– Change the dressing twice a day or as necessary given the amount of secretions^(11)^. – Change the dressing daily^(12,19)^. – Sanitize the peristomal skin with saline solution^(12,19)^. – Sanitize the peristomal skin with 3% hydrogen peroxide, soap and water, absorbent cotton, cotton buds, clean cloths and gauze^(15,20)^. – Identify signs of infection in the stoma^(22)^. – Use sterile water (obtained after boiling water for 20 minutes), a mirror to guide cleaning and gauze^(23)^.
	Hygienization of the endocannula^(1,11-23)^.	– Perform mechanical cleaning with sterile water or sterile saline solution^(11,23)^. – Clean with hydrogen peroxide^(17,23)^. – Clean the endocannula daily^(12,13,23)^. – Use lukewarm water and neutral detergent (with the exception of silicone cannula, where only saline solution is recommended)^(12)^. – Use brushes^(1,11,12)^ or gauze^(11)^. – Use a dishwasher for 90 minutes at 70ºC or use hot water, brushes and detergent, followed by complete drying^(17)^. – Clean with sodium bicarbonate solution, cloth or gauze and running water, boil the endocannula for 10 minutes and dry with a clean cloth after cooling^(19)^.
	Tracheostomy fixation^(11,12,14,15,21)^.	– Change the fixation with four hands (two people)^(11)^. – Change the fixation every day, keeping a gap of two fingers so as not to suffocate the patient^(12)^. – Change the fixation every day, keeping a gap of one finger between the fixator and the patient’s neck^(15)^.
	Airway humidification^(11,12,14,18,20)^.	– Perform airway nebulization daily, 1 to 5 times a day^(18)^.
	Emergency care^(12,15,16)^.	– Reinsert the cannula (in case of decannulation)^(15)^. – Remove the endocannula (in the event of a mucous plug), assess breathing, aspirate secretions and, if the patient does not improve, call the emergency services^(15)^. – Avoid removing the tracheostomy tube^(16)^.
	Stoma protection^(13)^.	– Protect the stoma with elements that prevent the entry of foreign bodies^(13)^.
	Nutrition^(14,18)^.	– Offering a balanced diet^(18)^.
	Management of cuff^(14,18)^.	No recommendations / adaptations.
	Hand hygiene before handling the tracheostomy^(14,16)^.	No recommendations / adaptations.
**Psychosocial**	Re-establishing the ability to communicate^(1,13,14,18,20,22,23)^.	– Instruct the patient about the esophageal voice^(1,13)^ and communication aids^(1)^.
	Social reintegration of the patient^(1,22)^.	– Ensure family support by developing strategies^(1)^. – Encourage the occupation of spaces and the adoption of common routines^(1)^.

## DISCUSSION

Only two of the studies included in the sample covered by this review were developed in Brazil, highlighting the lack of national research focusing on this issue, taking into account the context of our country and the Unified Health System. In general, there was a shortage of content on tracheostomy care in the home environment, especially those related to psychosocial and psychospiritual needs.

It can be seen that the problem affects patients with tracheostomies in home care all over the world; however, more national research on the subject is needed in Brazil. It is important to consider the size of the country, cultural diversity and access to health, financial resources and public health policies linked to this care in order to ensure a better quality of life for all those involved in the care process.

The patient must be understood as a whole, considering aspects related to their personal history, their habits, their way of seeing the disease, as well as the psychosocial context in which they are inserted, not just taking into account the biological demand of the disease that made them seek health care^(24)^. Thus, in order to discuss the results of this review, we chose to consider Wanda Horta’s view of BHN, which understands man as a holistic being and assigns nursing the role of ensuring that the individual - be it the patient, family or community - receives care that encompasses psychobiological, psychosocial and psychospiritual needs, in order to make them independent, by teaching self-care^(10)^.

Care must be personalized and adapted to each patient, since humans are dynamic beings, endowed with uniqueness, authenticity and individuality^(10)^. In this respect, the importance of considering the specificities of tracheostomized individuals is discussed, especially the elderly, as they present particularities resulting from age, the prevalence of chronic diseases and biological and social fragility^(25)^. In view of these characteristics, care for the elderly should be structured differently from the care provided to adults. However, the studies in this review did not take into account the specific characteristics of the elderly population. Only one article^(14)^ emphasized specific care for this population, but did not differentiate it from care for adults.

Among the care related to psychobiological needs, aspiration of tracheobronchial secretions stands out, cited by nine (64%) of the studies in this review. Airway suction is an intervention that consists of removing secretions from the lower respiratory tract by inserting a catheter with a negative pressure system, using aseptic techniques to keep the airways patent^(26,27)^. The selected studies recommended aseptic technique^(1,11,12,14,15,18,20,22,23)^, pre-oxygenation of the patient^(18)^, vacuum pressure of between 100 and 150 mmHg^(11,12)^, and a procedure duration of between 10 and 15 seconds^(11,12,23)^. Some studies^(12,20)^ have recommended humidifying the airways with saline solution through nebulization, in order to fluidize the tracheobronchial secretions and facilitate the aspiration procedure. Most of the recommendations identified for airway suctioning are in line with the American Association for Respiratory Care (AARC)^(5)^, with the exception of saline instillation proposed by one article^(23)^.

Although mandatory, airway suction does not lack complications^(28)^, thus requiring professionals who perform it to have specific and up-to-date knowledge^(26)^. The availability of good quality evidence on airway aspiration is still a major challenge for health professionals, as the published studies are supported by a low level of evidence^(28)^. In this context, transferring care associated with aspiration to patients and caregivers in the home environment becomes complex and challenging.

In a hospital environment, tracheostomized patients are cared for by a multi-professional healthcare team, which is able to identify possible complications more quickly and intervene appropriately in emergency scenarios, as well as having the appropriate equipment and materials^(5)^. However, despite the complex care, tracheostomy care can be carried out in the home environment^(5)^, as long as it is properly guided and adapted. Health education for hospital discharge is crucial for the safety of care that will be carried out by people who are not health professionals.

Regarding the hygiene care for the stoma and the tracheostomy tube itself, alternative and homemade tools have been suggested, such as toothbrushes^(11,23)^, clean cloths^(15)^, cotton buds^(15)^, gauze^(11,12,19,23)^, sodium bicarbonate solution^(15,19)^, simple soap^(15,19,20)^ and hydrogen peroxide^(15,17,19,23)^. In addition, one study^(23)^ provides instructions on preparing sterile water (boiled water for 20 minutes) and saline solution (2 teaspoons of salt in a liter of boiled water for 10 minutes), adapting the procedures to the context of the home^(23)^. There are many product recommendations for sanitizing the peristomal skin and endocannula, which highlights the need for experimental studies to recommend specific and safe products for such care.

The endocannula should be cleaned manually using brushes or gauze moistened with water or sterile saline every day^(11)^. Correct sanitization requires the patient to be seated to remove the endocannula and the gauze/cloths around the tracheostomy^(19)^. While cleaning is taking place, it is important to place gauze soaked in saline solution to prevent dust, for example, from entering the tracheostomy opening^(19)^. The endocannula should be carefully inserted again afterwards.

Fixing the tracheostomy is also a process that requires care, such as changing the lace every day, cleaning the contact area between the skin and the fixation device with soap and water, waiting for it to dry and checking that there is at least one finger of space between the lace/fixation tape and the patient’s neck^(15,23)^. There was a recommendation to change the fixation “four-handed”, i.e. with the help of another person^(11)^.

With regard to care related to psychosocial needs, the articles analyzed were limited to questions about the difficulty of clear verbal communication due to the tracheal stoma^(13)^; low self-esteem due to the dysmorphic perception of the appearance of the individual with the tracheostomy tube^(21)^; a strong tendency towards social isolation^(1)^; resistance to returning to daily activities^(1)^; physical and mental overload for caregivers/family members and the need for community support to reduce anxiety in care practices and to guarantee the autonomy and reintegration of tracheostomized individuals^(15)^. Although these issues are present in the literature, there has been no in-depth or more detailed explanation of how these impacts can be resolved through care in the home context.

The literature discusses the effects of tracheostomy on patients’ mental health, who often have mood swings, adjustment disorders and depression as a result of the aesthetic changes, difficulties in communication and social interaction, changes in eating and respiratory activity resulting from the procedure^(29)^. These factors not only result in a loss of self-esteem for the patient, but also hinder their recovery and autonomy^(29)^.

It is essential that health professionals are able to recognize the implications of tracheostomy on the patient’s quality of life and provide guidance to the individual/caregiver/family on the specific care needs of tracheostomized patients^(29)^. The lack of data exploring this niche of care makes it difficult for professionals to approach it in the period leading up to discharge and has a direct impact on the individual’s rehabilitation process.

Psychospiritual care was not reported in the studies in this review, which reveals the need to expand research into this topic. Although neglected in the studies, spirituality has a significant impact on an individual’s health and has been considered by the World Health Organization (WHO) as one of the essential factors for achieving quality of life^(30)^.

Spiritual care helps to improve self-acceptance and adaptation to the patient’s clinical condition^(31)^. The manifestation of spirituality, mainly linked to the subject’s inclusion in a faith community and interaction with participating members, has been shown to be beneficial in alleviating mental suffering by providing an environment in which the patient can be heard without judgment, included socially and have their concerns expressed^(31)^. Spirituality can also be a foundation for individuals in terms of their psychosocial needs, especially with regard to self-esteem and socialization^(31)^. Professionals often find it difficult to give instructions to the individual/family/caregiver about spiritual support, as they don’t cover all the individual’s needs^(31)^.

Some limitations should be considered in this study. The criterion of selecting studies only in English, Portuguese and Spanish may have limited the identification of more publications. The type of scoping review study does not require an assessment of the quality of the available evidence, which limits inferences about the quality of care and its clinical implications.

## CONCLUSION

The variation of interventions and contents identified in the present review regarding home care for tracheostomies, mainly addresses the psychobiological needs of human beings. Few studies presented care related to psychosocial needs, and there was a lack of care focused on the tracheostomized patient’s psychospiritual needs.

The findings allowed to identify that airway aspiration care in the home environment merits more clinical studies and systematic reviews. Its adaptation to the home setting must be supported by robust evidence, as it is a complex procedure and prone to complications. The transfer of care, including educational interventions and counter-referral actions, is consolidated as an intervention by the hospital team, and is the most relevant action for the success and safety of the tracheostomized patient in the home environment.

This review encourages the development of more studies on the subject, especially with regard to the adaptations of care at home, taking into account all the needs of the individual, with the aim of subsidizing the planning of hospital discharge for tracheostomized patients.
