# Supplementary material for: Tracheostomy care for adults and the elderly in the home environment: a scoping review
Source: Rev Esc Enferm USP. 2024 Aug 2;58:e20240028. doi: 10.1590/1980-220X-REEUSP-2024-0028en (PMC11299533; doi:10.1590/1980-220X-REEUSP-2024-0028en)
Supplement: Translation

# Translation: Cuidados com traqueostomia em adultos e idosos no ambiente domiciliar: revisão de escopo

**DOI:** 10.1590/1980-220X-REEUSP-2024-0028pt

**Authors:** Aldenora Laísa Paiva de Carvalho Cordeiro, Jade Alycia Ribeiro e Santos, Ana Clara Leite Barroso, Miguir Terezinha Vieccelli Donoso, Luciana Regina Ferreira Pereira da Mata, Tânia Couto Machado Chianca

**Affiliations:** 1 Universidade Federal de Minas Gerais, Escola de Enfermagem, Departamento de Enfermagem Básica, Belo Horizonte, MG, Brasil.; 2 Universidade Federal de Minas Gerais, Escola de Enfermagem, Belo Horizonte, MG, Brasil.

**Keywords:** Traqueostomia, Assistência Domiciliar, Alta do Paciente, Enfermagem

## Abstract

**Objetivo:**

Mapear o conhecimento científico sobre cuidados com a traqueostomia de adultos e idosos realizados pelo indivíduo, família ou cuidador no ambiente domiciliar.

**Método:**

Revisão de Escopo, conduzida em fevereiro de 2023, segundo a metodologia do *Joanna Briggs Institute*. A questão norteadora foi: quais e como devem ser realizados os cuidados com a traqueostomia de adultos/idosos conduzidos pelo indivíduo/família/cuidador no ambiente domiciliar? Foram considerados estudos publicados em português, inglês e espanhol. As bases de dados consultadas foram: Lilacs; Medline, via PubMed; Cinahl; Cochrane Library; PEDro; Embase; Scopus e Web of Science.

**Resultados:**

Foram identificados 2158 artigos, dos quais 81 foram lidos na íntegra e 14 incluídos na revisão. Os principais cuidados identificados contemplavam necessidades psicobiológicas: aspiração de vias aéreas, troca de fixação da traqueostomia, limpeza da endocânula e higienização da pele periestomal. Quanto às necessidades psicossociais foram identificados: incentivos à comunicação e à autonomia. Não houve recomendações de cuidados relativos às necessidades psicoespirituais.

**Conclusão:**

Os achados priorizam cuidados biológicos e poucos estudos apresentaram o detalhamento sobre como realizar tais cuidados no domicílio.

## INTRODUÇÃO

A traqueostomia, procedimento cirúrgico comumente realizado em ambiente hospitalar, consiste na abertura da parede anterior do pescoço e da traqueia, onde se insere uma cânula que permite a comunicação com o ambiente externo e garante a manutenção da permeabilidade das vias aéreas^(1)^. É comumente indicada para indivíduos que necessitam de suporte ventilatório e para reversão de obstrução prolongada ou permanente das vias aéreas superiores^(2)^.

Indivíduos traqueostomizados enfrentam diversos desafios de caráter biológico e psicossocial, especialmente, na alta hospitalar^(1)^. A ausência de orientações sobre cuidados no domicílio aumenta a chance de complicações que podem resultar em reinternações^(1,2)^.

Alta precoce e não capacitação do paciente e família/cuidador para o cuidado domiciliar foram reconhecidos como uns dos principais fatores de risco para readmissões hospitalares de indivíduos com traqueostomia^(3,4)^. Visto que no período pós-operatório e na alta hospitalar o cuidado na maioria das vezes é ofertado de forma fragmentada, o apoio e o fornecimento de instruções para realização do cuidado domiciliar tornam-se imprescindíveis^(1)^.

O planejamento da alta hospitalar para uma efetiva transição do cuidado garante a continuidade e a segurança do paciente traqueostomizado, prevenindo incidentes críticos e reospitalizações^(2)^. Esse planejamento deve ser compreendido como um processo contínuo que se inicia na admissão hospitalar e deve envolver a tríade indivíduo-família-comunidade^(1,3)^.

No entanto, embora a ciência^(1-4)^ reforce a importância e a necessidade de ensino e aconselhamento à pessoa e/ou família/cuidador, não estão claros quais e como devem ser realizados os cuidados com a traqueostomia de adultos/idosos no contexto do ambiente domiciliar. A *American Association for Respiratory Care* (AARC) publicou em 2022 um *Clinical Practice Guidelines* ^(5)^ sobre a aspiração de vias aéreas, mas ainda não há em suas publicações diretrizes que contemplem as especificidades dos cuidados com a traqueostomia em domicílio.

Em uma pesquisa preliminar realizada em janeiro de 2023 na *Medical Literature Analysis and Retrieval System Online* (Medline), no Banco de Dados Cochrane de Revisões Sistemáticas e na Síntese de Evidências do *Joanna Briggs Institute* (JBI), não foram encontrados estudos de revisão com a abordagem dos cuidados domiciliares. Apenas uma revisão de escopo^(2)^ foi encontrada, contudo, o foco das intervenções de enfermagem mapeadas foi o autocuidado com a traqueostomia, sem a descrição sobre como realizar os cuidados no ambiente domiciliar^(2)^, o que justifica a proposição deste estudo. Assim, o objetivo desta revisão de escopo foi mapear o conhecimento científico sobre cuidados com a traqueostomia de adultos e idosos realizados pelo indivíduo, família ou cuidador no ambiente domiciliar. A expectativa da presente revisão é clarificar quais os cuidados necessários e como estes devem ser realizados.

## MÉTODO

### Tipo de Estudo

Trata-se de uma revisão de escopo. O protocolo desta revisão foi registrado na plataforma *Open Science Framework* (OSF)^(6)^ e seguiu a metodologia proposta pelo JBI, descrita no JBI Reviewer’s Manual^(7)^. Para a redação, foram seguidas as recomendações do *‘Preferred Reporting Items for Systematic Reviews and Meta-Analyses for Scoping Reviews”* (PRISMA-ScR)^(8)^, específico para revisões de escopo.

### Questão Norteadora

A questão norteadora foi estruturada de acordo com o mnemônico “PCC”, de População, Conceito e Contexto^(7)^: P- pacientes adultos e idosos traqueostomizados; C – cuidado realizado pelo próprio paciente, família ou cuidador; C– ambiente domiciliar. Assim, a questão norteadora desta revisão foi: “quais e como devem ser realizados os cuidados com a traqueostomia em adultos/idosos conduzidos pelo indivíduo/família/cuidador no ambiente domiciliar?”

O conceito e contexto “cuidado realizado pelo próprio paciente ou família/cuidador no ambiente domiciliar”, está ancorado na definição do *Medical Subject Headings* (MeSH) da Medline para o termo “*Home Nursing”*, que refere aos cuidados de enfermagem prestados a uma pessoa no domicílio, que podem ser realizados pelo próprio paciente, família, amigo ou a um cuidador, devidamente treinados.

### Critérios de Elegibilidade

Os critérios de seleção foram estabelecidos com base na questão norteadora, considerando a estratégia PCC. Foram considerados estudos com as seguintes características: a) quanto à população – estudos realizados com indivíduos com traqueostomia definitiva ou temporária, com idade de 18 anos ou superior; b) quanto ao conceito – estudos que avaliaram ou descreveram cuidados passíveis de serem conduzidos pelo próprio indivíduo ou família/cuidador; c) quanto ao contexto – estudos que contemplassem os cuidados realizados no ambiente domiciliar.

Foram incluídos estudos publicados nas línguas portuguesa, inglesa e espanhola; pesquisas primárias com delineamentos quantitativos, qualitativos e de métodos mistos, estudos de revisão e de opinião clínica. Não foi estabelecido limite de tempo para a publicação dos estudos incluídos. Foram excluídos estudos que não apresentaram qual seria o cenário de realização dos cuidados com a traqueostomia.

### Estratégia de Busca e Seleção dos Estudos

A estratégia de busca foi elaborada por um profissional bibliotecário e foram consideradas as bases de dados: Literatura Latino-Americana e do Caribe em Ciências da Saúde (Lilacs); Medline, via PubMed; *Cumulative Index to Nursing and Allied Health Literature* (Cinahl), Cochrane Library, Google Schoolar, *Physiotherapy Evidence Database* (PEDro), Embase, Scopus e Web of Science. As três últimas bases foram acessadas por meio do portal CAPES (Coordenação de Aperfeiçoamento de Pessoal de Nível Superior) do Brasil. Foram organizados conjuntos de termos controlados e não controlados relacionados à população (P), conceito (C) e contexto (C). Foi realizada também a busca na literatura cinzenta por meio das seguintes bases: Catálogo de Teses e Dissertações da Capes; Portal Brasileiro de Publicações e Dados Científicos em Acesso Aberto (OASISBR) e *OpenGrey*.

O quadro 1, a seguir, apresenta a estratégia de busca utilizada para cada base.

**Quadro 1:** Estratégias de busca aplicadas a cada base de dados, Belo Horizonte, MG, Brazil, 2023.

BASE	ESTRATÉGIA
LILACS	(traqueostomia OR tracheostomy OR traqueostomía OR trachéostomie) AND (“Assistência Domiciliar” OR “Home Nursing” OR “Atención Domiciliaria de Salud” OR “Soins à domicile” OR “Ambiente Domiciliar” OR “Home Environment” OR “Ambiente en el Hogar” OR “Enfermagem Domiciliar” OR “Home Health Nursing” OR “Cuidados de Enfermería en el Hogar” OR “Soins infirmiers à domicile” OR “Home Care”) AND ( db:(“LILACS” OR “BDENF” OR “IBECS” OR “BINACIS” OR “campusvirtualsp_brasil” OR “CUMED” OR “INDEXPSI” OR “MedCarib” OR “SES-SP” OR “SMS-SP” OR “SOF” OR “colecionaSUS”))
MEDLINE VIA PUBMED	(Tracheostomy) AND (“Home Nursing” OR “Home Environment” OR “Home Health Nursing” OR “Home Care”)
CINAHL	(Tracheostomy) AND (“Home Nursing” OR “Home Environment” OR “Home Health Nursing” OR “Home Care”)
COCHRANE	(Tracheostomy) AND (“Home Nursing” OR “Home Environment” OR “Home Health Nursing” OR “Home Care”)
GOOGLE SCHOLAR	(Tracheostomy OR Traqueostomia) AND (“Home Nursing” OR “Assistência Domiciliar”)
PEDro	(Tracheostomy)
EMBASE (Via Portal Capes)	(Tracheostomy) AND (‘Home Care’ OR ‘Home Environment’)
SCOPUS (Via Portal Capes)	(Tracheostomy) AND (“Home Nursing” OR “Home Environment” OR “Home Health Nursing” OR “Home Care”)
WEB OF SCIENCE (Via Portal Capes)	(Tracheostomy) AND (“Home Nursing” OR “Home Environment” OR “Home Health Nursing” OR “Home Care”)
**LITERATURA CINZENTA**	**ESTRATÉGIA**
Catálogo de teses e dissertações da capes	(Traqueostomia) AND (“Assistência Domiciliar”)
OASISBR	(Traqueostomia) AND (“Assistência Domiciliar”)
OpenGrey	(Tracheostomy) AND (“Home Nursing”)

O levantamento dos estudos nas bases de dados foi realizado em fevereiro de 2023 por dois pesquisadores, de forma independente. A pré-seleção consistiu na leitura dos títulos e resumos, considerando os critérios de elegibilidade.

Foi utilizado o *software* de revisão *Rayyan Qatar Computing Research Institute* (*Rayyan* QCRI). Esse programa proporcionou às pesquisadoras a execução da seleção de estudos de forma sistematizada e rápida, além de permitir excluir as duplicatas e fazer o cegamento entre os pesquisadores, o que assegurou a confiabilidade e a precisão metodológica para esta etapa^(9)^.

Após a pré-seleção, prosseguiu-se com a leitura na íntegra dos artigos por dois pesquisadores, de forma independente. Em caráter complementar, houve uma busca manual por citação (*handsearch*) na lista de referências dos artigos selecionados para a leitura na íntegra. Um terceiro pesquisador foi necessário nos casos em que houve divergência entre os outros dois pesquisadores. As discordâncias foram resolvidas com discussão entre os três pesquisadores até que se chegasse a um consenso.

### Extração e Análise de Dados

A extração dos dados foi conduzida por dois pesquisadores, de forma independente, com a finalidade de diminuir o risco de viés de aferição. O instrumento para extração dos dados foi elaborado pelos pesquisadores e contemplou os seguintes dados: autor(es), ano de publicação, origem do estudo, objetivos, população e tamanho da amostra, tipo de estudo, resultados e detalhes destes, principais descobertas relacionadas à pergunta da revisão de escopo. Quando necessário, os autores dos trabalhos foram contatados para levantamento de informações e de detalhes acerca dos dados.

Na segunda etapa, os dados sobre os cuidados foram agrupados e resumidos de acordo com as categorias referentes às necessidades humanas básicas^(10)^ psicobiológicas, psicossociais e psicoespirituais. Todos os pesquisadores participaram da interpretação e síntese dos dados. Os dados foram apresentados de forma tabular e analisados de forma narrativa.

## RESULTADOS

Um total de 2256 artigos foi identificado nas bases de dados e 14 estudos foram recuperados a partir da busca manual nas referências dos estudos incluídos e na literatura cinzenta, conforme apresentado na Figura 1. Após a remoção das duplicatas (n = 1181), 996 estudos foram excluídos durante a leitura dos resumos, por não responderem à pergunta da revisão, visto que versavam sobre uma população menor de 18 anos (n = 475), ou não se enquadravam no contexto e no conceito estabelecidos nos critérios de elegibilidade da pesquisa (n = 521). Foi realizada a leitura completa de 93 artigos, dentre os quais 14 compuseram a amostra desta revisão de escopo.

**Figura 1:** Fluxograma PRISMA-ScR de seleção das publicações – Belo Horizonte, MG, Brasil, 2024.

A figura 1 ilustra o fluxo do processo de busca e seleção dos artigos incluídos na revisão de escopo.

O período de publicação dos estudos variou do ano de 1985 a 2022 e, em relação ao idioma, 11 (79%) foram publicados em inglês, um (7%) em espanhol e dois (14%) em português. Quanto ao local dos estudos, três (21,4%) foram desenvolvidos nos Estados Unidos; dois (21,4%) no Irã; dois (21,4%) na Turquia; dois (21,4%) no Brasil; um (7,1%) na Inglaterra, Chile, Nova Zelândia, Bélgica e Índia. O quadro 2 apresenta a caracterização dos estudos.

**Quadro 2:** Caracterização dos estudos incluídos na revisão de escopo. Belo Horizonte, MG, Brazil, 2023. Fonte: Dados da pesquisa, 2023.

Título	Ano/ País	Objetivo	Tipo de estudo	Principais resultados
[E1] Uma revisão sistemática das experiências de pacientes e cuidadores com traqueostomia^(1)^.	2018, Nova Zelândia.	Compreender o conhecimento atual relacionado à experiência e à qualidade de vida de adultos vivendo com traqueostomia e seus cuidadores, para melhorar o cuidado.	Revisão sistemática.	Cinco temas principais emergiram da pesquisa: (1) cuidado, suporte e gerenciamento de uma traqueostomia; (2) fala e comunicação; (3) bem-estar e qualidade de vida; (4) desfiguração e imagem corporal; e (5) estigma e isolamento social.
[E2] Descrição e manejo do paciente traqueostomizado em internação domiciliar: experiência no Complexo Assistencial Doutor Sótero do Rio^(11)^.	2022, Chile.	Descrever o perfil e o manejo de pacientes com traqueostomia em hospitalização domiciliar.	Descritivo, retrospectivo.	O objetivo de ensinar o manejo e a educação em traqueostomia foi aplicado em 87 dos 96 pacientes participantes e contemplou cuidados gerais com traqueostomia e estoma, conhecimento do dispositivo e manejo de urgências e de situações de decanulação.
[E3] Cuidados para a prevenção de complicações em pacientes traqueostomizados^(12)^.	2019, Brasil.	Analisar as evidências de cuidados para a prevenção de complicações em pacientes traqueostomizados.	Revisão integrativa.	Fatores relacionados à aspiração endotraqueal, à limpeza do tubo e da pele e à educação em saúde representaram as principais estratégias que minimizam o risco de complicações.
[E4] As necessidades de aprendizagem dos pacientes laringectomizados^(13)^.	1992, Brasil.	Relatar as necessidades de aprendizagem de pacientes laringectomizados atendidos em um hospital universitário.	Qualitativo, do tipo observação-participante.	As necessidades de aprendizagem identificadas referem-se à aquisição de informações e habilidades relacionadas com: função da laringe, doença, objetivos e consequências da cirurgia e dos tratamentos; possibilidades de exercer atividades profissionais, de lazer e sexuais; possibilidade de treinamento da voz esofágica; imagem corporal alterada; desenvolvimento de habilidades para o autocuidado com a traqueostomia.
[E5] Educação sobre cuidados com traqueostomia e seu efeito no conhecimento e na sobrecarga de cuidadores de pacientes idosos: um estudo quase experimental^(14)^.	2019, Turquia.	Investigar o efeito do treinamento para manejo da traqueostomia destinado a cuidadores de pacientes idosos nos níveis de sobrecarga e de conhecimento dos cuidadores.	Quase-experimental.	O treinamento aumentou o nível de conhecimento do cuidador e diminuiu a sobrecarga de cuidado. Atitudes e habilidades a serem adquiridas devem ser explicadas em uma linguagem compreensível para pacientes e cuidadores e ensinadas por métodos de demonstração durante o período da internação.
[E6] Desenvolvendo o programa de alívio da ansiedade no tratamento da traqueostomia por meio de educação e apoio (T-CARES)^(15)^.	2014, Estados Unidos.	Descrever o desenvolvimento e os resultados do programa T-CARES, desenvolvido em resposta às altas taxas de readmissão de pacientes com câncer de cabeça e pescoço que receberam alta com uma traqueostomia.	Quase-experimental.	O programa T-CARES mostra-se promissor na redução da ansiedade e no aumento da competência de aspiração em cuidadores domiciliares de um familiar com uma nova traqueostomia.
[E7] Ganhando experiência ao longo do tempo: percepção dos cuidadores familiares sobre pacientes com traqueostomia em atendimento domiciliar^(16).^	2021, Irã.	Explicar as vivências de cuidadores e familiares de pacientes com traqueostomia acerca do cuidado do paciente em casa.	Qualitativo, análise de conteúdo.	As categorias de vivências identificadas no estudo incluem: necessidade de treinamento; necessidade de receber suporte de cuidados; desafios de cuidados; fardo; experiência, esperança e satisfação interior.
[E8] A desinfecção da tubulação de ventilação mecânica é necessária em casa^(17)^?	2006, Bélgica.	Determinar o nível de limpeza e esterilidade do circuito de ventilação domiciliar (HCV) em pacientes em ventilação domiciliar e a eficiência do protocolo para limpeza do HVC recomendado.	Experimental.	Foram apresentadas recomendações como: o uso de uma máquina de lavar louça duas vezes por mês para lavar todas as peças de tubagens, circuitos e máscaras de ventilação e a limpeza das cânulas de traqueostomia com peróxido de hidrogênio ou através de um banho quente com escova e detergente. A descontaminação só é indicada quando o tubo é visualmente sujo e/ou quando os pacientes traqueostomizados são sensíveis às infecções do trato respiratório.
[E9] Cuidando de pacientes com traqueostomia no domicílio: um estudo descritivo e transversal para avaliar as práticas de cuidado em saúde e a sobrecarga do cuidador^(18)^.	2019, Turquia.	Determinar as práticas de cuidados de saúde e a sobrecarga dos cuidadores familiares de pacientes com traqueostomia que vivem em casa.	Descritivo, transversal.	O comportamento de cuidado dos cuidadores foi avaliado nas categorias: conhecimento sobre atendimento de emergência, número de instrumentos para atendimento, frequência de visita domiciliar, experiência em situação de emergência nos últimos seis meses e atividades da vida diária do paciente.
[E10] Conselhos de enfermagem sobre cuidados domiciliares para pacientes com câncer de cabeça e pescoço na Índia^(19)^.	2006, Índia.	Instruir pacientes, familiares e cuidadores acerca de procedimentos e cuidados necessários com a boca, traqueostomia e sonda nasogástrica.	Opinião clínica.	Cuidados com a traqueostomia incluem a troca e limpeza do tubo interno; a limpeza ao redor do local da traqueostomia e a troca diária de curativos; busca por sinais de infecção na região da boca e do estoma; além do preparo de soluções para higienização das estruturas envolvidas no dispositivo.
[E11] Cuidados de longo prazo do paciente com traqueostomia^(20)^.	2005, Estados Unidos.	Discutir os componentes de um programa de tratamento de traqueostomia de longo prazo.	Opinião Clínica.	A educação do paciente deve iniciar antes da inserção da traqueostomia e incluir: anatomia básica das vias aéreas; justificativa para uso de traqueostomia; descrição da operação do tubo; sinais e sintomas de desconforto respiratório; técnica de aspiração; limpeza e manutenção do tubo; avaliação e limpeza do local do estoma; ressuscitação cardiopulmonar; decanulação de emergência e procedimentos de reinserção; procedimento de troca de tubo; uso de equipamentos e suprimentos.
[E12] Autocuidado com traqueostomia: The Nottingham System^(21)^.	1992, Inglaterra.	Descrever o Sistema Nottingham que visa ensinar autocuidado com a traqueostomia de forma escalonada.	Descritivo.	São descritos cuidados como: reconhecer a necessidade de limpar e trocar o tubo; remoção, limpeza e inserção do tubo interno; preparação do equipamento para troca do tubo externo; aplicação de fitas; remoção, limpeza e inserção do tubo externo; cuidados com a pele.
[E13] Efeito de vídeos para instrução domiciliar na qualidade de vida de pacientes traqueostomizados: ensaio clínico randomizado^(22)^.	2015, Irã.	Verificar o efeito de vídeo instrucional na qualidade de vida de pessoas com traqueostomia.	Quase-experimental.	Os cuidados diários apresentados foram: banho, barbear, realizar aspiração, realizar o curativo na pele periestomal, limpar o tubo de traqueostomia, saber identificar sintomas de infecção no local da traqueostomia, saber como se comunicar com outras pessoas e como se apresentar em público.
[E14] Ajudando um paciente laringectomizado a voltar para casa^(23)^.	1985, Estados Unidos.	Descrever cuidados com pacientes laringectomizados, que vão desde comunicação ao ensino do autocuidado.	Opinião Clínica.	Foram descritos cuidados que visam auxiliar na recuperação da independência e autonomia dos pacientes, tais como treinamento da comunicação, remoção e limpeza diária da cânula, aconselhamento nutricional e outros procedimentos de autocuidado.

Os cuidados foram agrupados de acordo com as necessidades humanas básicas (NHB) propostas por Wanda Horta (1979)^(10)^. Cuidados relacionados às necessidades psicobiológicas foram citados em todos os estudos da amostra com destaque para: aspiração de vias aéreas (nove/ 64%); higienização do estoma e da endocânula (12/ 85%); e troca de curativos e dos cadarços de fixação (nove/ 64%). Dentre os 14 estudos, sete (50%) também descreveram cuidados que atendem às necessidades psicossociais, com ênfase para o reestabelecimento da capacidade de comunicação oral do paciente, por meio da aprendizagem de voz esofágica e de outros dispositivos auxiliares de fala. A necessidade de reinserção social do paciente, considerando sua autonomia e apoio da família e de outros grupos sociais, esteve presente em dois (14%) estudos. Cuidados relacionados às necessidades psicoespirituais não foram identificados.

O quadro 3 apresenta os cuidados identificados na literatura agrupados em NHB, bem como as recomendações sobre como realizar esses cuidados.

**Quadro 3:** Síntese dos artigos incluídos sobre cuidados de traqueostomia em adultos e idosos no ambiente domiciliar (n = 14). Belo Horizonte, MG, Brazil, 2023. Fonte: Dados da pesquisa, 2023.

NHB	Cuidados identificados com a traqueostomia no ambiente domiciliar	
	Cuidado	Recomendações sobre como realizar o cuidado
**Psicobiológica**	Aspiração de secre-ções^(1,11,12,14,15,18,20,22,23)^.	– Utilizar técnica asséptica^(11)^. – Realizar pré–oxigenação do paciente^(18)^. – Utilizar pressão do vácuo entre 100 a 150 mmHg^(11,12)^. – Realizar o procedimento entre 10 a 15 segundos^(11,12,23)^. – Instilar soro fisiológico^(23)^. – Utilizar aspirador portátil^(1)^. – Realizar o procedimento diariamente, de1 a 5 vezes ao dia^(18)^.
	Cuidados com o estoma: higienização e troca de curativos^(11-16,18-23)^.	– Realizar a troca do curativo duas vezes ao dia ou de acordo com a necessidade frente à quantidade de secreções^(11)^. – Realizar a troca de curativo diariamente^(12,19)^. – Higienizar a pele periestoma com soro fisiológico^(12,19)^. – Higienizar a pele periestoma com peróxido de hidrogênio a 3%, água e sabão, algodão, cotonetes, panos limpos e gazes^(15,20)^. – Identificação de sinais de infecção no estoma^(22)^. – Utilizar água estéril (obtida após ferver água por 20 minutos), um espelho para guiar a limpeza e gazes^(23)^.
	Higienização da endocânula^(1,11-23)^.	– Realizar limpeza mecânica com água estéril ou soro fisiológico estéril^(11,23)^. – Realizar a limpeza com peróxido de hidrogênio^(17,23)^. – Realizar limpeza da endocânula diariamente^(12,13,23)^. – Utilizar água morna, detergente neutro (com exceção da cânula de silicone, na qual recomenda–se a utilização apenas de soro fisiológico)^(12)^. – Utilizar escovas^(1,11,12)^ ou gazes^(11)^. – Utilizar máquina de lavar louças por 90 minutos à 70ºC ou utilizar água quente, escovas e detergente, seguida por secagem completa^(17)^. – Realizar a limpeza com solução de bicarbonato de sódio, pano ou gaze e água corrente, ferver a endocânula por 10 minutos e após esfriar, secar com pano limpo^(19)^.
	Fixação da traqueostomia^(11,12,14,15,21)^.	– Realizar a troca da fixação à quatro mãos (duas pessoas)^(11)^. – Realizar a troca da fixação diariamente, mantendo uma folga de dois dedos para não sufocar o paciente^(12)^. – Realizar a troca da fixação diariamente, mantendo uma folga de um dedo de espaço entre o fixador e o pescoço do paciente^(15)^.
	Umidificação de vias aéreas^(11,12,14,18,20)^.	– Realizar nebulização de vias aéreas diariamente, 1 a 5 vezes ao dia^(18)^.
	Cuidados de emergência^(12,15,16)^.	– Reinserir a cânula (em caso de decanulação)^(15)^. – Remover a endocânula (em caso de tampão mucoso), avaliar a respiração, aspirar secreções e, caso o paciente não melhore, ligar para o serviço de emergência^(15)^. – Evitar a remoção da cânula de traqueostomia^(16)^.
	Proteção do estoma^(13)^.	– Proteger o estoma com elementos que impeçam a entrada de corpos estranhos^(13)^.
	Nutrição^(14,18)^.	– Oferecer dieta balanceada^(18)^.
	Gerenciamento do balonete (*cuff*)^(14,18)^.	Ausência de recomendações / adaptações.
	Higienização das mãos antes de manusear a traqueostomia^(14,16)^.	Ausência de recomendações / adaptações.
**Psicossocial**	Reestabelecimento da capacidade de comunicação^(1,13,14,18,20,22,23)^.	– Instruir o paciente quanto a voz esofágica^(1,13)^ e auxiliares de comunicação^(1)^.
	Reinserção social do paciente^(1,22)^.	– Garantir o apoio da família, por meio do desenvolvimento de estratégias^(1)^. – Incentivar a ocupação de espaços e a adoção de rotinas comuns^(1)^.

## DISCUSSÃO

Nesta revisão apenas dois artigos da amostra foram desenvolvidos no Brasil, o que evidencia a carência de pesquisas nacionais com enfoque nesta temática, que considerem o contexto do nosso país e do Sistema Único de Saúde. De forma geral, observou-se a escassez de conteúdo sobre cuidados com a traqueostomia no ambiente domiciliar, principalmente, aqueles relacionados às necessidades psicossociais e psicoespirituais.

Nota-se que o problema afeta pacientes com traqueostomia em cuidado domiciliar em todo o mundo, entretanto, no Brasil são necessárias mais pesquisas nacionais sobre a temática. É importante considerar a dimensão do país, a diversidade cultural e de acesso à saúde, os recursos financeiros e as políticas de saúde pública vinculadas a esses cuidados a fim de assegurar maior qualidade de vida a todos os envolvidos no processo de cuidar.

O paciente deve ser compreendido como um todo, considerando aspectos relacionados à sua história pessoal, seus hábitos, sua forma de ver a doença, além do contexto psicossocial em que está inserido, não levando em conta somente a demanda biológica da doença que o fez buscar por atendimento de saúde^(24)^. Assim, para discutir os resultados desta revisão, optou-se por considerar a visão de Wanda Horta sobre as NHB, que compreende as pessoas como seres holísticos e atribui à Enfermagem a função de garantir que o indivíduo - seja ele o paciente, família ou comunidade - receba cuidados que englobam necessidades psicobiológicas, psicossociais e psicoespirituais, de forma a torná-lo independente, por meio do ensino do autocuidado^(10)^.

Esses cuidados devem ser personalizados e adaptados para cada paciente, visto que as pessoas são seres dinâmicos, dotado de unicidade, autenticidade e individualidade^(10)^. Neste aspecto, discute-se a importância de se considerar as especificidades dos indivíduos traqueostomizados, sobretudo, os idosos, por apresentarem particularidades decorrentes da idade, da prevalência de doenças crônicas e das fragilidades biológicas e sociais^(25)^. Frente a essas características, o cuidado com o idoso deve ser estruturado de forma diferenciada à assistência prestada à pessoa adulta. No entanto, nos estudos dessa revisão não foram consideradas as especificidades da população idosa. Somente um artigo^(14)^ enfatizou os cuidados específicos para essa população, mas não os diferenciou dos cuidados com adultos.

Entre os cuidados relacionados às necessidades psicobiológicas, destaca-se a aspiração de secreções traqueobrônquicas, citado por nove (64%) estudos dessa revisão. A aspiração de vias aéreas é uma intervenção que consiste na remoção de secreções do trato respiratório inferior, por meio da inserção de um cateter com sistema de pressão negativa, com técnica asséptica para manter as vias aéreas pérvias^(26,27)^. Nos estudos selecionados, foi recomendada a técnica asséptica^(1,11,12,14,15,18,20,22,23)^, a pré-oxigenação do paciente^(18)^, pressão do vácuo entre 100 a 150 mmHg^(11,12)^, e duração do procedimento entre 10 a 15 segundos^(11,12,23)^. Alguns estudos^(12,20)^ recomendaram a umidificação das vias áreas com solução salina, por meio de nebulização, com o intuito de fluidificar as secreções traqueobrônquicas e facilitar o procedimento da aspiração. A maioria das recomendações identificadas para a aspiração de vias aéreas está em consonância com a *American Association for Respiratory Care* (AARC)^(5)^, com exceção da instilação salina proposta por um artigo^(23)^.

Embora mandatória, a aspiração de vias aéreas não é isenta de complicações^(28)^, exigindo assim, que os profissionais que a executam possuam conhecimentos específicos e atualizados^(26)^. A disponibilidade de evidências de boa qualidade sobre a aspiração de vias aéreas ainda representa um grande desafio para os profissionais da saúde, pois os estudos publicados são sustentados por baixo nível de evidência^(28)^. Nesse âmbito, a transferência de cuidados associados à aspiração para pacientes e cuidadores no ambiente domiciliar, torna-se algo complexo e desafiador.

Em um ambiente hospitalar, a assistência ao paciente traqueostomizado é realizada pela equipe multiprofissional de saúde, a qual é capaz de identificar mais rapidamente possíveis complicações e intervir adequadamente em cenários emergenciais, além de dispor de equipamentos e materiais adequados^(5).^ No entanto, apesar da assistência complexa, os cuidados com a traqueostomia são passíveis de serem realizados no ambiente domiciliar^(5)^, desde que sejam orientados e adaptados de forma apropriada. A educação em saúde para a alta hospitalar é determinante para a segurança dos cuidados que serão conduzidos por pessoas que não são profissionais de saúde.

Quanto aos cuidados para a higiene do estoma e da própria cânula de traqueostomia, foram sugeridos ferramentas alternativas e caseiras, como escovas de dentes^(11,23)^, panos limpos^(15)^, cotonetes^(15)^, gazes^(11,12,19,23)^, solução de bicarbonato de sódio^(15,19)^, sabão simples^(15,19,20)^ e peróxido de hidrogênio^(15,17,19,23)^. Além disso, um estudo^(23)^ fornece instruções sobre o preparo de água estéril (água fervida durante 20 minutos) e de solução salina (2 colheres de chá de sal em um litro de água fervida por 10 minutos), adaptando os procedimentos ao contexto do domicílio^(23)^. Há muitas recomendações de produtos para a higienização da pele periestoma e das endocânulas, o que evidencia a necessidade de estudos experimentais que recomendem produtos específicos e seguros para tais cuidados.

A endocânula deve ser limpa manualmente com uso de escovas ou gazes úmidas com água ou soro estéril todos os dias^(11)^. A higienização correta exige que o paciente esteja sentado para a retirada da endocânula e das gazes/panos ao redor da traqueostomia^(19)^. Enquanto ocorre a limpeza, é importante colocar gazes embebidas com solução salina para evitar que entre poeira, por exemplo, na abertura da traqueostomia^(19)^. Em seguida, deve-se inserir novamente a endocânula com cuidado.

A fixação da traqueostomia também é um processo que exige cuidados, como trocar o cadarço diariamente, limpar a região de contato entre a pele e o dispositivo de fixação com água e sabão, esperar secar e verificar se há pelo menos um dedo de espaço entre o cadarço/fita de fixação e o pescoço do paciente^(15,23)^. Houve a recomendação de realizar a troca da fixação a “quatro mãos”, ou seja, com a ajuda de outra pessoa^(11)^.

No que tange aos cuidados relacionados com as necessidades psicossociais, os artigos analisados se limitam a abordar questões sobre a dificuldade de comunicação verbal clara, em função do estoma traqueal^(13)^; baixa autoestima devido à percepção dismórfica da aparência do indivíduo com a cânula de traqueostomia^(21)^; forte tendência ao isolamento social^(1)^; resistência ao retorno às atividades cotidianas^(1)^; sobrecarga física e mental dos cuidadores/familiares e a necessidade de apoio da comunidade para redução da ansiedade na práticas dos cuidados e para a garantia de autonomia e reinserção do indivíduo traqueostomizado^(15)^. Embora essas questões estejam presentes na literatura, não houve um aprofundamento, nem uma explicação mais detalhada sobre como esses impactos podem ser solucionados por meio de cuidados no contexto domiciliar.

A literatura discorre sobre as repercussões da traqueostomia na saúde mental dos pacientes, os quais muitas vezes apresentam variações de humor, transtornos de ajustamento e depressão resultantes das mudanças estéticas, dificuldades de comunicação e interação social, alterações na alimentação e na atividade respiratória decorrentes do procedimento^(29)^. Esses fatores resultam não só na perda de autoestima do paciente como também dificultam sua recuperação e sua autonomia^(29)^.

É essencial que os profissionais de saúde estejam aptos a reconhecerem as implicações da traqueostomia na qualidade de vida do paciente e que forneçam orientações ao indivíduo/cuidador/família sobre as necessidades de cuidado específicos ao paciente traqueostomizado^(29)^. A ausência de dados que explorem esse nicho de cuidados dificulta a abordagem do profissional no período que antecede a alta e reverbera diretamente no processo de reabilitação do indivíduo.

Os cuidados psicoespirituais não foram relatados nos estudos desta revisão, o que revela a necessidade de ampliar as investigações nessa temática. Apesar de negligenciada nos estudos, a espiritualidade traz impactos relevantes para a saúde do indivíduo e tem sido considerada pela Organização Mundial de Saúde (OMS) como um dos fatores essenciais para se alcançar qualidade de vida^(30)^.

O cuidado espiritual ajuda a melhorar a auto aceitação e a adaptação à condição clínica do paciente^(31)^. A manifestação da espiritualidade, principalmente atrelada à inserção do sujeito em uma comunidade de fé e à interação com os membros participantes, mostrou-se benéfica para aliviar o sofrimento mental ao proporcionar um ambiente em que o paciente possa ser ouvido sem julgamentos, incluído socialmente e ter suas preocupações expressas^(31)^. A espiritualidade pode ainda ser alicerce para o indivíduo quanto às suas necessidades psicossociais, principalmente no que diz respeito à autoestima e socialização^(31)^. Muitas vezes, os profissionais sentem dificuldades em elaborar instruções ao indivíduo/família/cuidador sobre suporte espiritual, não contemplando todas as necessidades do indivíduo^(31)^.

Algumas limitações devem ser consideradas neste estudo. A definição do critério de seleção de trabalhos apenas nos idiomas inglês, português e espanhol pode ter limitado a identificação de mais publicações. O tipo de estudo revisão de escopo não requer a avaliação da qualidade da evidência disponível, o que limita inferir sobre a qualidade dos cuidados e suas implicações clínicas.

## CONCLUSÃO

A variabilidade de intervenções e conteúdos identificados nesta revisão quanto aos cuidados domiciliares com a traqueostomia, em sua maioria, contempla as necessidades psicobiológicas do ser humano. Poucos estudos apresentaram cuidados relacionados às necessidades psicossociais, e ausência de cuidados voltados para a necessidade psicoespiritual do paciente traqueostomizado.

Foi possível identificar que os cuidados com a aspiração de vias aéreas no ambiente domiciliar carecem de mais estudos clínicos e revisões sistemáticas. Suas adaptações para o contexto domiciliar devem estar sustentadas por evidências robustas, por ser um procedimento complexo e passível de complicações. A transferência do cuidado, incluindo as intervenções educativas e as ações de contrarreferência, consolidam-se como uma intervenção da equipe hospitalar, sendo a ação mais relevante para o sucesso e segurança do paciente traqueostomizado no ambiente domiciliar.

A partir dessa revisão, incentiva-se o desenvolvimento de mais estudos sobre a temática, principalmente, quanto às adaptações do cuidado no domicílio, contemplando todas as necessidades do indivíduo e com o intuito de subsidiar o planejamento da alta hospitalar do paciente traqueostomizado.
